# Is virtual reality useful for pain management in patients who undergo medical procedures?

**DOI:** 10.31744/einstein_journal/2019MD4837

**Published:** 2019-05-15

**Authors:** Daniel Melecchi de Oliveira Freitas, Viviane Souto Spadoni

**Affiliations:** 1 Hospital Moinhos de Vento, Porto Alegre, RS, Brazil.; 2 Hospital de Clínicas de Porto Alegre, Porto Alegre, RS, Brazil.

**Keywords:** Virtual reality, Pain management, Virtual reality exposure therapy, Realidade virtual, Manejo da dor, Terapia de exposição à realidade virtual

## Abstract

Pain management is a complex medical issue, and many efforts have been done to develop new non-pharmacological therapies. Virtual reality is a technology apparatus that make an interaction between human and virtual environment through an hardware (usually a headset) linked to a computer or a mobile, by using a software. Additionally, this virtual setting can be adapted to any type of scenario. Thus, it is plausible that the software used should be personalized depending on patient’s experiences and expectations. The use of virtual reality as a medical tool for pain relief or decrease analgesics use by promoting a cognitive distraction is a low cost and promising instrument for pain management in patients who undergo medical procedures.

## RESUMO

O manejo da dor é uma questão médica complexa, e muitos esforços têm sido feito para que novas terapias não farmacológicas sejam desenvolvidas. A realidade virtual é um aparato tecnológico que promove interação entre humanos e ambiente virtual, por meio de *hardware* (geralmente fones de ouvido), conectado a computador ou celular, e de *software*. Além disso, este ambiente virtual pode ser adaptado para qualquer tipo de cenário. Portanto, é plausível que o *software* utilizado seja personalizado, dependendo da experiência do paciente e de suas expectativas. O uso da realidade virtual como ferramenta médica para alívio da dor ou redução do uso de analgésicos, por meio de distração cognitiva, é um instrumento promissor e de baixo custo para manejo da dor em indivíduos submetidos a procedimentos médicos.

## INTRODUCTION

Although pain mechanism is more commonly related to inflammatory response and nerve injury, this is a complex distressing feeling not completely understood.^(^ ^1^ ^)^ Nociception is not only associated to pain location and type of stimulus, but also correlated with a subjective and personal pain threshold that is very difficult to evaluate before medical procedures.^(^ ^2^ ^)^ Cognitive distraction have been studied as a way of relieving pain in several situations with the intention of changing the manner it is perceived by the patient. Such a complex network is composed not only by somatosensory system but also affective system that can modulate pain intensity and quality. Moreover to make patients not pay attention to painful stimulus has been also studied as a powerful method for change pain perception.^(^ ^3^ ^)^

Recently, pain management became an important worldwide health issue. Few months ago, the Center for Disease Control and Prevention (CDC) sent an important notification letter alerting about opioid dependence and increasing in costs regarding the use of these substances in primary care patients in the U.S.^(^ ^4^ ^)^ The agency emphasized the need of development of new non- pharmacologic therapies to reduce opioid overuse and avoid side effects.^(^ ^4^ ^)^In this setting, new technologies have been explored to assist medical community in this issue.

### Virtual reality and augmented reality

**Figure 1:**
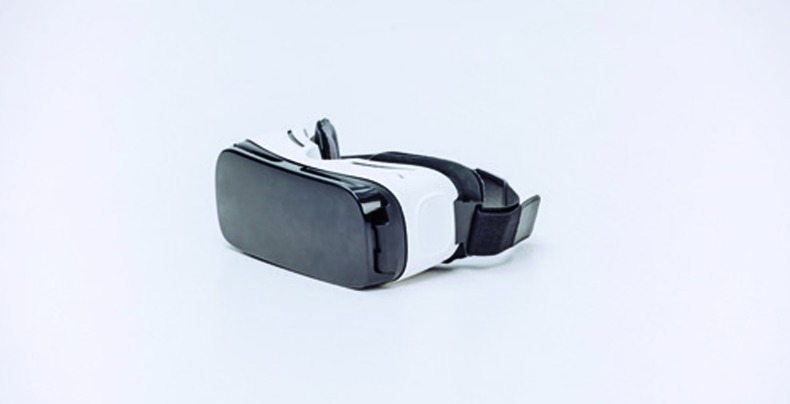
Virtual reality headset

**Figure 2:**
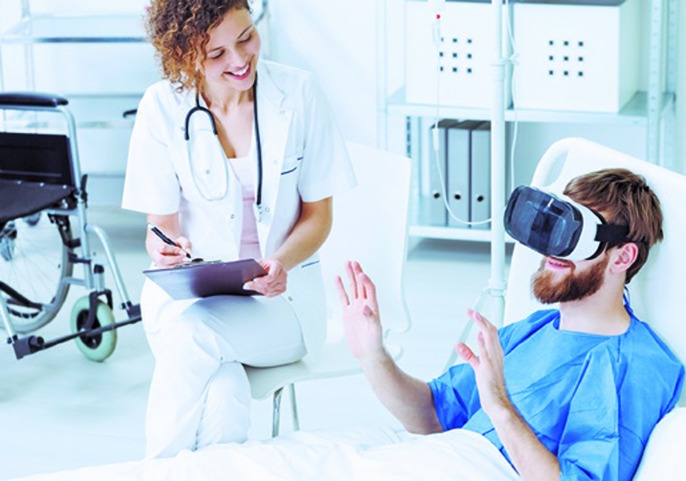
The use of virtual reality headset during medical procedures

Virtual reality (VR) systems are composed by hardware (headset, glasses, gloves, computers or mobile devices) and software that provide a VR environment in multiple contexts (Figure 1). The environment can be a place, like a hospital, a classroom, a meeting room, or even a hologram that can interact directly with the user. While VR build a complete holographic environment, in the augmented reality (AR) the holograms are mixed with the real world around of the subject. Additionally, with the exponential technological evolution in the last two decades, VR has become an interesting tool for learning, cognitive training, rehabilitation and also treatment of patients with some medical disorders^(^ ^5^ ^-^ ^7^ ^)^ (Figure 2). The development of new graphics, rendering, animation, voice recognition and artificial intelligence provided a new era in the human-machine interaction.

## DISCUSSION

Several health professionals have incorporated technology in their practice. Given the recent exponential development in computer systems, the usage of VR and AR became an interesting way for pain relief.^(^ ^1^ ^,^ ^5^ ^-^ ^10^ ^)^

Previous studies that analyzed VR on pain management have demonstrated mixed results.^(^ ^5^ ^,^ ^6^ ^)^ For example, Das et al., after randomizing burn victims using or not VR associated with analgesics during dressing changes, found that subjects who experimented the technology associated with medication, presented lower average pain scores compared with the Control Group.^(^ ^7^ ^)^ Likewise, Mott et al., who studied the AR associated with analgesics in a prospective randomized trial, found similar results. In that study 42 children with acute burns were allocated to have AR experience during dressing change. The authors demonstrated that AR group had significant lower pain scores when compared with the Control Group.^(^ ^8^ ^)^ Another trial that evaluated VR in patients with various chronic pain conditions demonstrated pain relief during VR-sections in different degrees in all patients who were enrolled.^(^ ^9^ ^)^ Recently, Moon et al., after randomizing 40 patients before undergoing trans-urethral urological procedures under spinal anesthesia found that patients using VR during surgery were more satisfied than those who were sedated with midazolan. In that study, surgeons and anesthesiologists were also more satisfied when used technology.^(^ ^10^ ^)^ Conversely, Walker et al., after analyzing the use of VR for pain relief during cystoscopy, failed to demonstrate any difference in anxiety and pain scores between the groups.^(^ ^11^ ^)^

The implications of VR/AR use during medical procedures are an interesting area of study. Given that pain is an extremely complex process, this is also related to previous experiences, emotional status and the type of painful stimulus, the use of cognitive distraction emerges as a promising technology. The reason for conflicting results relies probably on heterogeneity of groups and singularity of each individual. Nonetheless, a strong factor that favors VR/AR is it cheapness and absence of side effects. As pain threshold is an individual and subjective issue, the VR/AR usage during medical procedures should be personalized. To interview the patient to analyze his or her personality, anxieties, fears, thoughts and feelings is an interesting method to select what virtual experience would be more helpful in reducing the way pain is processed and perceived. In this context, more studies are needed to select the best patient for this technology as well as the type of hardware and virtual environment during each specific medical procedure.
